# Supplementary material for: MetaRibo-Seq measures translation in microbiomes
Source: Nat Commun. 2020 Jun 29;11:3268. doi: 10.1038/s41467-020-17081-z (PMC7324362; doi:10.1038/s41467-020-17081-z)
Supplement: Supplementary file 10 — Supplementary Data 7 [file 41467_2020_17081_MOESM10_ESM.zip › File2/Confidence_High_Taxonomy/407166_out.krona.html]

Javascript must be enabled to view this page.

members
magnitude
magnitudeUnassigned
count
unassigned
taxon
rank

407166\_out

5

2
5
superkingdom

5
976
phylum

class
200643
5

order
5
171549

family
2005525
5

5
375288
genus

823
5
species

SRS075984\_contig\_number\_12270SRS104327\_contig\_number\_540SRS142542\_contig\_number\_5121SRS148253\_contig\_number\_1584SRS148817\_contig\_number\_8503
